# One-pot synthesis of cyanohydrin derivatives from alkyl bromides via incorporation of two one-carbon components by consecutive radical/ionic reactions

**DOI:** 10.3762/bjoc.10.12

**Published:** 2014-01-14

**Authors:** Shuhei Sumino, Akira Fusano, Hiroyuki Okai, Takahide Fukuyama, Ilhyong Ryu

**Affiliations:** 1 Department of Chemistry, Graduate School of Science, Osaka Prefecture University, Sakai, Osaka 599-8531, Japan

**Keywords:** alkyl bromide, carbon monoxide, cyanohydrin, ethyl cyanoformate, multicomponent, radical reaction

## Abstract

The consecutive radical/ionic reaction consisting of radical formylation of alkyl bromides and nucleophilic addition of a cyanide ion was investigated, which gave moderate to good yields of cyanohydrin derivatives in one-pot.

## Introduction

**Scheme 1:**
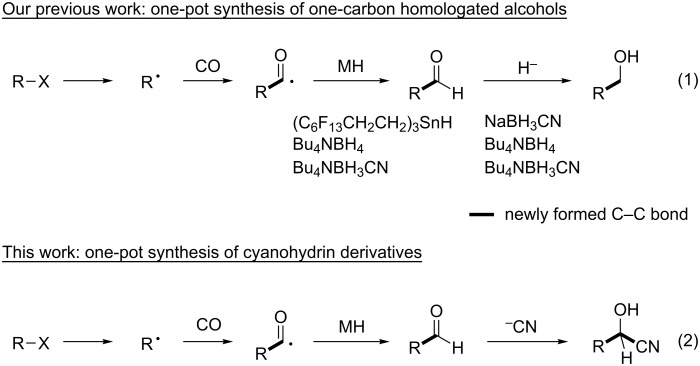
Sequential radical formylation and derivatization.

Radical carbonylation reactions have been recognized as a versatile tool for the synthesis of a wide variety of carbonyl compounds [1–4]. In 1990, we demonstrated that aldehydes can be prepared from alkyl or aromatic halides and CO under typical radical chain reaction conditions using tributyltin hydride and AIBN [5–6]. Under the reaction conditions where a catalytic amount of fluorous tin hydride and an excess amount of sodium cyanoborohydride were used, initially formed aldehydes can be converted into hydroxymethylated compounds in one-pot [7–9], since borohydride acts not only as the reagent for the regeneration of tin hydride [10–13] but also as the reagent for aldehyde reduction. Later on we found that borohydride reagents can also serve as radical mediator delivering hydrogen to the radical centre [14], thus we developed a hydroxymethylation method using Bu_4_NBH_4_ and a radical initiator [15–17]. Recent work in collaboration with Dennis Curran has revealed that, with the use of NHC-borane [18], hydroxymethylation of aromatic iodides can be attained [19]. All these reactions consist of the combination of radical formylation with CO and ionic hydride reduction by hydride reagents (Scheme 1, reaction 1). During the course of our study on borohydride-mediated radical hydroxymethylation of alkyl halides with CO, we found that cyanohydrin was obtained as a byproduct when Bu_4_NBH_3_CN was used as a radical mediator [15], which led us to investigate the one-pot synthesis of cyanohydrins based on radical formylation. Thus, we thought that the two step radical/ionic reactions can be extended to the consecutive C–C bond forming reactions.

Cyanohydrins are important subunits frequently found in biologically active compounds and are also versatile building blocks for further synthetic transformations [20–21]. The common method to obtain cyanohydrins is the reaction of aldehydes with a cyanide sources such as TMSCN [22–23], ethyl cyanoformate [24–26] or acyl cyanide [27–28]. We provide here an efficient one-pot method for the synthesis of cyanohydrin derivatives via consecutive radical/ionic C–C bond forming reaction of alkyl bromides, CO and ethyl cyanoformate (Scheme 1, reaction 2).

## Results and Discussion

**Scheme 2:**
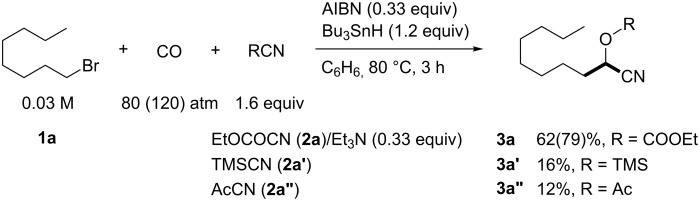
Examination of cyanide source.

We examined AIBN-induced radical formylation of 1-bromooctane (**1a**) with Bu_3_SnH under 80 atm of CO pressure in the presence of a cyanide source (Scheme 2). Under the employed conditions, the reaction using TMSCN (**2a'**) was slow, which gave 16% of **3a'** and 51% of nonanal. The use of AcCN (**2a''**) also gave **3a''** but only in 12% yield. However, when ethyl cyanoformate (**2a**) was used together with Et_3_N [29], the cyanohydrin **3a** was obtained in 62% yield. When we used higher CO pressure such as 120 atm, the yield of **3a** increased to 79%.

We examined various alkyl bromides **1** in the present radical/ionic three-component coupling reaction (Table 1). Primary alkyl bromides **1b–e** containing a chlorine atom, an ester group, a cyano group, or a phenyl group worked well to give the corresponding cyanohydrin derivatives **3b–e** in good yields (Table 1, entries 2–5). The reaction of secondary and tertiary alkyl bromides **1f–i** also proceeded well to give the corresponding cyanohydrins **3f–i** in good yields (Table 1, entries 6–9). The reaction using cyclopropylmethyl bromide (**1j**) afforded the lowest yield of cyanohydrin **3j**, which possessed an olefin structure arising from the ring-opening of a cyclopropylcarbinyl radical (Table 1, entry 10) [30–31].

**Table 1:** Three-component coupling reaction leading to cyanohydrin derivatives.

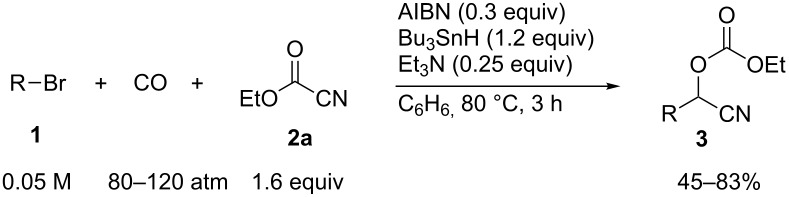				

entry	alkyl bromide	CO (atm)	product	yield^a^ (%)

1^b^	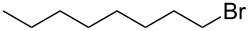 **1a**	120	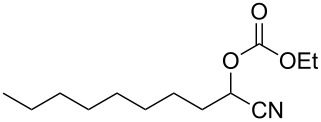 **3a**	79
2	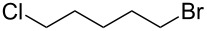 **1b**	80	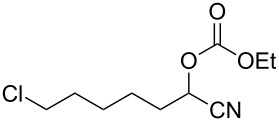 **3b**	60
3	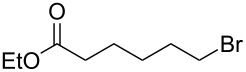 **1c**	80	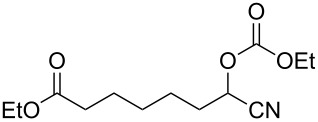 **3c**	83
4	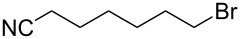 **1d**	120	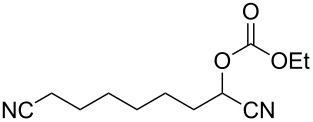 **3d**	76
5	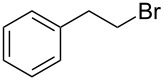 **1e**	120	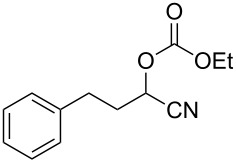 **3e**	61
6	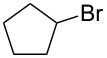 **1f**	120	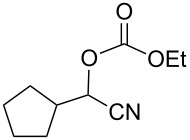 **3f**	61
7	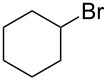 **1g**	120	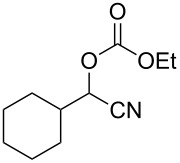 **3g**	74
8	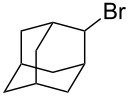 **1h**	120	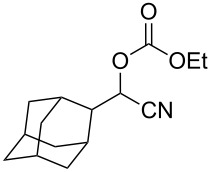 **3h**	73
9	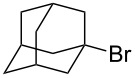 **1i**	110	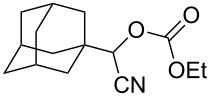 **3i**	82
10	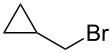 **1j**	110	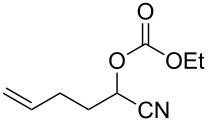 **3j**	45

^a^Isolated yield after flash chromatography on SiO_2_. ^b^0.03 M.

## Conclusion

In summary, we have demonstrated a three-component coupling reaction comprising alkyl bromides **1**, CO and ethyl cyanoformate (**2a**) in the presence of Bu_3_SnH, AIBN, and Et_3_N, which gave moderate to good yields of cyanohydrin derivatives **3**. This protocol represents a one-pot method [32–33] based on radical carbonylation and ionic cyanation.

## Experimental

Typical procedure for radical/ionic three-component coupling reaction leading to cyanohydrin derivatives 1-cyanononyl ethyl carbonate (**3a**) [34] (Table 1, entry 1): A mixture of 1-bromooctane (**1a**, 96.6 mg, 0.5 mmol), ethyl cyanoformate (**2a**, 79.3 mg, 0.8 mmol), tributyltin hydride (174.6 mg, 0.6 mmol), triethylamine (13.2 mg, 0.13 mmol), and AIBN (24.6 mg, 0.15 mmol) in C_6_H_6_ (17 mL) were placed in a 100 mL stainless steel autoclave. The reaction mixture was degassed 3 times with 10 atm of CO and charged with 90 atm of CO at −40 °C (MeCN–dry ice bath). Then the autoclave was allowed to warm to room temperature, which caused the pressure gauge to indicate 120 atm. Then the reaction was conducted at 80 °C for 3 h. After cooling to room temperature, the reaction mixture was concentrated and purified by silica gel flash chromatography (hexane/EtOAc 97:3) to afford **3a** (95.3 mg, 79%). ^1^H NMR (CDCl_3_, 500 MHz) δ 5.18 (t, *J* = 6.8 Hz, 1H), 4.4–4.2 (m, 2H), 2.0–1.9 (m, 2H), 1.6–1.5 (m, 2H), 1.4–1.2 (m, 13H), 0.88 (t, *J* = 6.9 Hz, 3H); ^13^C NMR (CDCl_3_, 125 MHz) δ 153.56, 116.51, 65.27, 64.66, 32.31, 31.68, 29.12, 28.99, 28.71, 24.34, 22.53, 14.05, 13.93.

## References

[1] Ryu I, Sonoda N (1996). Angew Chem, Int Ed Engl.

[2] Ryu I, Sonoda N, Curran D P (1996). Chem Rev.

[3] Chatgilialoglu C, Crich D, Komatsu M, Ryu I (1999). Chem Rev.

[4] Ryu I (2001). Chem Soc Rev.

[5] Ryu I, Kusano K, Ogawa A, Kambe N, Sonoda N (1990). J Am Chem Soc.

[6] Ryu I, Kusano K, Masumi N, Yamazaki H, Ogawa A, Sonoda N (1990). Tetrahedron Lett.

[7] Gupta V, Kahne D (1993). Tetrahedron Lett.

[8] Ryu I, Niguma T, Minakata S, Komatsu M, Hadida S, Curran D P (1997). Tetrahedron Lett.

[9] Matsubara H, Yasuda S, Sugiyama H, Ryu I, Fujii Y, Kita K (2002). Tetrahedron.

[10] Corey E J, Suggs W (1975). J Org Chem.

[11] Giese B, González-Gómez J A, Witzel T (1984). Angew Chem, Int Ed Engl.

[12] Stork G, Sher P M (1986). J Am Chem Soc.

[13] Curran D P, Hadida S, Kim S-Y, Luo Z (1999). J Am Chem Soc.

[14] Ryu I, Uehara S, Hirao H, Fukuyama T (2008). Org Lett.

[15] Kobayashi S, Kawamoto T, Uehara S, Fukuyama T, Ryu I (2010). Org Lett.

[16] Kobayashi S, Kinoshita T, Kawamoto T, Wada M, Kuroda H, Masuyama A, Ryu I (2011). J Org Chem.

[17] Kawamoto T, Ryu I (2012). Chimia.

[18] Curran D P, Solovyev A, Makhlouf Brahmi M, Fensterbank L, Malacria M, Lacôte E (2011). Angew Chem, Int Ed.

[19] Kawamoto T, Okada T, Curran D P, Ryu I (2013). Org Lett.

[20] Gregory R J H (1999). Chem Rev.

[21] Brunel J-M, Holmes I P (2004). Angew Chem, Int Ed.

[22] Lidy W, Sundermeyer W (1973). Chem Ber.

[23] Evans D A, Truesdale L K, Carroll G L (1973). J Chem Soc, Chem Commun.

[24] Poirier D, Berthiaume D, Boivin R P (1999). Synlett.

[25] Berthiaume D, Poirier D (2000). Tetrahedron.

[26] Tian S-K, Deng L (2001). J Am Chem Soc.

[27] Hoffmann H M R, Ismail Z M, Hollweg R, Zein A R (1990). Bull Chem Soc Jpn.

[28] Okimoto M, Chiba T (1996). Synthesis.

[29] Baeza A, Nájera C, de Garcia Retamosa M, Sansano J M (2005). Synthesis.

[30] Bowry V W, Ingold K U (1991). J Am Chem Soc.

[31] Newcomb M (1993). Tetrahedron.

[32] Suga S, Yamada D, Yoshida J-i (2010). Chem Lett.

[33] Yoshida J-i, Saito K, Nokami T, Nagaki A (2011). Synlett.

[34] Khan N H, Agrawal S, Kureshy R I, Abdi S H R, Sadhukhan A, Pillai R S, Bajaj H C (2010). Catal Commun.

